# Testing the EQ-HWB-9 with seven-day versus today recall periods in dementia: variability and recall adherence under health fluctuations

**DOI:** 10.1007/s11136-026-04432-4

**Published:** 2026-11-06

**Authors:** Maresa Buchholz, Lidia Engel, Feng Xie, Bernhard Michalowsky

**Affiliations:** 1 Patient-Reported Outcomes & Health Economics Research German Center for Neurodegenerative Diseases (DZNE), Site Rostock/ Greifswald, Ellernholzstrasse 1-2, 17489 Greifswald, Germany; 2 Monash University Health Economics Group, School of Public Health and Preventive Medicine, Monash University, 553 St Kilda Rd, Melbourne, VIC 3004 Australia; 3 Department of Economics and Business Administration, University of Applied Sciences Stralsund, Stralsund, Germany; 4 Centre for Health Economics and Policy Analysis, McMaster University, Hamilton, ON Canada

**Keywords:** Dementia, Health fluctuation, Patient-reported outcome measures, Psychometrics, Health-related quality of life, EQ-HWB, Recall periods

## Abstract

**Purpose:**

To compare a “7-DAY” and “TODAY” recall period of the EQ-HWB-9 in people living with dementia (PlwD) with regard to day-to-day health changes (health fluctuations) and recall period adherence.

**Methods:**

In this mixed-methods study, PlwD-caregiver dyads from German primary care were randomized to complete the EQ-HWB-9 (self- and proxy-proxy report) using either a "7-day" or “today” recall period over a two-week time period on day 1, day seven and day 14. In addition, caregivers recorded daily health fluctuations over 14 days via a diary. Health fluctuations were examined in relation to EQ-HWB-9 score variability and recall adherence, which was assessed via semi-structured caregiver interviews. Quantitative analyses included recall group comparisons, effect sizes, and variance analyses.

**Results:**

Seventy-four dyads were included (PlwD mean age 77 years; 41.9% female; caregiver mean age 71 years; 71.6% female). Proxy EQ-HWB-9 scores were higher (worse health) than self-reports and higher with the "7-day" versus “today” recall. Caregivers reported at least one health fluctuation in 92% of PlwD; fluctuations occurred in 41% of daily assessments. Greater diary-documented fluctuation was associated with higher EQ-HWB-9 score variability. Recall adherence differed by health fluctuation status: caregivers of PlwD with no to low health fluctuations favored TODAY, whereas those of PlwD with high health fluctuations favored the 7-DAY recall time frame.

**Conclusions:**

No single recall period appears optimal for all PlwD. Recall period selection should consider individual health fluctuation patterns to improve the performance of HRQoL assessments in dementia.

**Supplementary Information:**

The online version contains supplementary material available at https://doi.org/10.1007/s11136-026-04432-4.

## Plain Language summary

People living with dementia (PlwD) often experience recurrent, often unpredictable, day-to-day changes in their health, with some days better (e.g. better memory), others worse (e.g. aggressive behaviour), so-called health fluctuations. Measuring the quality of life in PlwD has become an essential goal in the treatment and research of dementia. Nevertheless, it is unknown how quality-of-life questionnaires can capture these day-to-day changes in PlwD across different recall periods. In our study, we compared a "7-day" recall period with a “today” recall period using the EQ-HWB-9 questionnaire, an instrument of health and well-being. PlwD and their caregivers completed the questionnaire over 2 weeks, on days 1, 7, and 14. At the same time, caregivers kept a daily diary documenting whether the PlwD’s health had improved, worsened, or stayed the same compared to the previous day. We found that health fluctuations were very common: caregivers reported to perceive at least one change in most of PlwD, which are often related to exhaustion, depression and cognitive changes. We also found that caregivers who document no to low health fluctuations are more likely to use the today recall period than those who report greater day-to-day changes in PlwD. These caregivers often adhere to the 7-day recall time frame. We conclude that the choice of recall periods matters. In conditions like dementia, where health can change frequently, selecting an appropriate recall period is essential for accurately capturing quality of life.

**Supplementary Information:**

The online version contains supplementary material available at https://doi.org/10.1007/s11136-026-04432-4.

## Introduction

Degenerative diseases rarely follow a steady, linear disease progression. Instead, individuals with chronic conditions often experience recurrent and unpredictable day-to-day changes in their health, commonly referred to as health fluctuations [1–3]. People living with dementia (PlwD) are some of those who frequently describe alternating periods of “better” and “worse” days, marked by enhanced cognition, motivation, and activity on good days and memory decline, irritability, or behavioural disturbances on bad days [4]. Cognitive variability has been examined in clinical studies [5, 6], emphasizing that cognitive fluctuations remain one of the most complex and least understood symptoms in dementia. Sun et al. [7] found that cognitive variability was associated with reduced capacity for daily activities and diminished health-related quality of life (HRQoL), underscoring the importance of considering short-term fluctuations in HRQoL research [4].

Prior studies have identified a recurring challenge in the use of generic preference-based HRQoL instruments, where respondents often fail to adhere to the intended recall periods and prefer various recall time frames due to their health condition [8]. This was particularly evident in the EQ-5D, which asks about “your health today” (Recall period “Today”) [9]. Although the EQ-5D is valid for use among individuals with moderate cognitive and functional impairment [10–13], its one-day recall time frame may not adequately reflect the overall health experience of patients who face frequent daily changes, thereby affecting both the validity and the reliability of responses. This was supported by a recent study by Michalowsky et al. [14], demonstrating that PlwD frequently experienced health fluctuations, on average, every second day. These fluctuations were primarily triggered by issues with memory, mobility, concentration, sleep, pain, and usual activities, affecting the HRQoL assessment using the EQ-5D-5​L and resulting in a higher variation in EQ-5D-5​L utility and non-adherence to the recall period. This warranted further research to evaluate whether extended recall periods could capture health fluctuations more accurately. Moreover, there is limited clarity on which specific dimensions are most influenced by symptom fluctuations, especially when considering broader constructs of wellbeing that go beyond health. The EQ Health and Well-being (EQ-HWB) is a new measure, specifically designed to be applicable across health and social care settings, reflecting aspects of life relevant to people with long-term and complex conditions. Importantly, the EQ-HWB uses a “7-day” recall period, which may enhance its ability to represent overall health in individuals experiencing recurrent symptom fluctuations, such as those living with dementia. This extended recall frame, alongside its multidimensional scope, may result in more valid and reliable assessments of HRQoL in populations affected by daily variability.

However, evidence about whether the EQ-HWB captures health state fluctuations different to those measures with a “Today” recall period is currently lacking. Therefore, this study aimed to close these gaps by using a daily health fluctuation diary and administering the EQ-HWB-9 questionnaire with its original “7-DAY” versus an experimental “TODAY” recall period, evaluating the variability of both recall periods, and recall period adherence.

## Methods

### Study design

This exploratory, observational study employed a mixed-methods approach to investigate (i) the day-to-day variability in HRQoL, as documented by informal caregivers (hereinafter referred to as “caregivers”) and PlwD (dyads) using a daily paper-based diary across 14 days, and (ii) the influence of these fluctuations on score variability and (iii) recall-period adherence when assessing HRQoL through the EQ-HWB-9 across two randomly assigned groups (based on dyads of PlwD and their caregivers) where the EQ-HWB-9 was administered with the original “7-DAY” recall period (GROUP 1) and an experimental recall period of “TODAY” (GROUP 2). Randomization was performed using a pre-defined allocation sequence to ensure an equal distribution of participant across both groups (1:1).

**Fig. 1:**
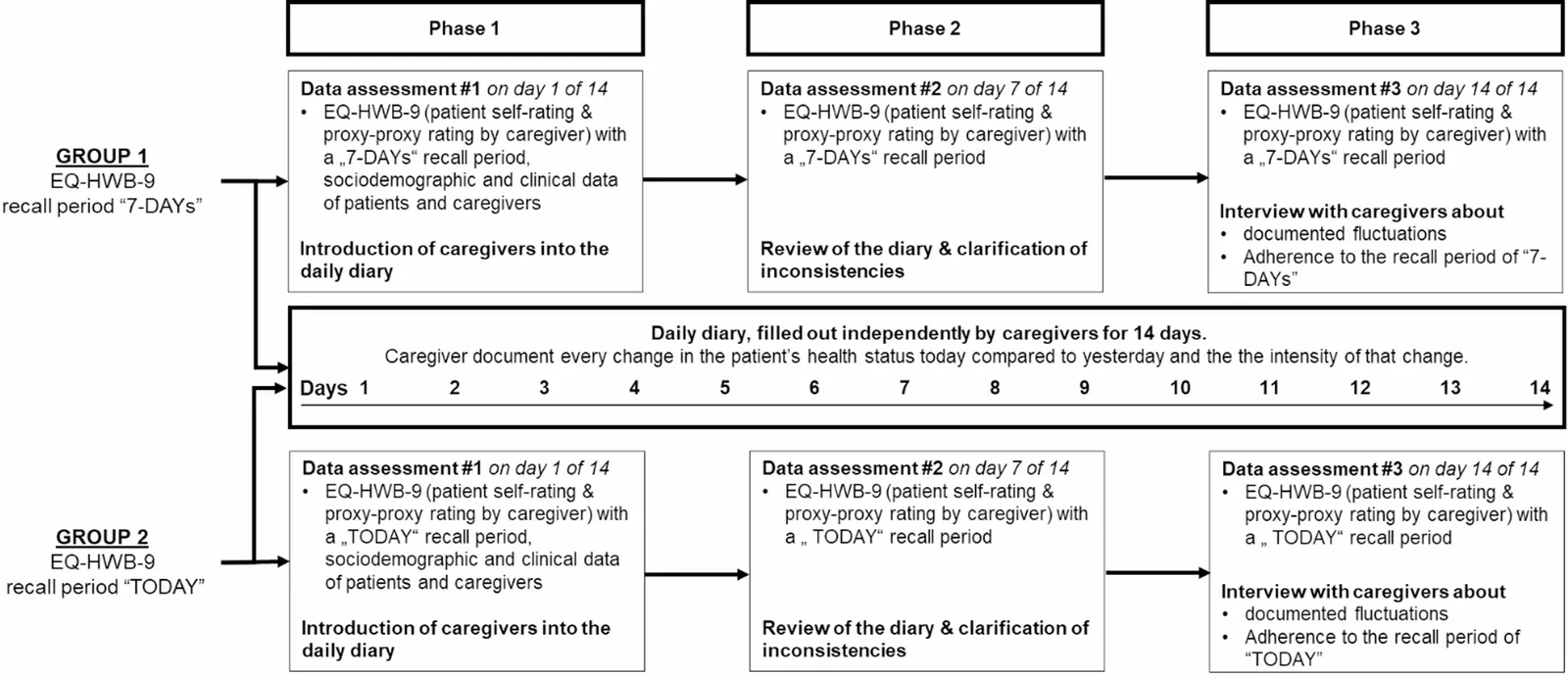
Study design, data assessment and instrument administration

The EQ-HWB-9 questionnaires were administered as a self- (by PlwD) and proxy-proxy version (by caregivers) (1st) on day 1, after starting the daily health fluctuation diary, (2nd) on day 7, and (3rd) on day 14, after completing the daily health fluctuation diary (see Fig. 1). Caregivers provided proxy-proxy ratings reflecting their own perception of the PlwD’s health and wellbeing, rather than proxy-person ratings estimating how patients would rate themselves [9]. The assigned recall period was specified for each EQ-HWB-9 domain. PlwD and caregivers were interviewed one after the other by the trained study nurse, without the other person being present. Finally, the caregivers were interviewed immediately after completing the daily diary and the last EQ-HWB-9 administration, using semi-structured interviews to determine whether they adhered to the given recall periods of “7 DAYS” (GROUP 1) or “TODAY” (GROUP 2).

The study design is demonstrated in Fig. 1.

### Setting and sample

Participant dyads, consisting of PlwD and their caregivers, were recruited from German primary care settings between May 2023 and December 2024. Recruitment was conducted through trained study nurses. Eligibility required a positive dementia screening using the DemTect tool (score ≤ 8) [15] or a formal dementia diagnosis by a treating physician (GP, neurologist, or psychiatrist). Caregivers were eligible if they lived with the PlwD together or visited the PlwD daily. Eligible participants were given study information and asked to provide written informed consent. Ethical approval was obtained from the local ethics committee at the University Medicine Greifswald (reference number BB 030/23).

Given the exploratory nature of the study, no formal sample size calculation was conducted. The sample size was informed by a previous study [14] in a similar population.

### Data assessment

The baseline and follow-up surveys were conducted via face-to-face interviews with the PlwD and the caregivers at the dyads’ homes. The paper-pencil diary was daily self-completed by caregivers.

#### Demographic and clinical data

PlwD’ demographic, including age, sex, living situation (alone vs. not alone) and the following clinical variables were assessed at baseline: cognitive impairment according to the Mini-Mental State Examination (MMSE) [16, 17], depression according to the Geriatric Depression Scale (GDS) [18], deficits in daily living activities according to the Bayer Activities of Daily Living Scale (B-ADL), comorbidity according to the Charlson Comorbidity Index (CCI) [19] and caregiver burden by the Zarit Burden Interview [20].

#### EQ-HWB-9

The EQ-HWB-9, a measure of health and well-being with a 7-day recall period, was explicitly developed for caregivers and care recipients to evaluate interventions in health, public health, and social care, including their impact on patients, social care users, and caregivers. The EQ-HWB was recently evaluated in caregivers of PlwD, demonstrating sufficient psychometric properties [21]. The EQ-HWB-9 (formerly EQ-HWB-S) is a concise, short-form instrument derived from the 25-item EQ-HWB [10], covering the following nine dimensions: mobility, daily activity, exhaustion, loneliness, cognition, anxiety, sadness/depression, control, and pain. Each domain has one question with five response-level options [22] (Supplementary File 1). At present, there is no German value set for the EQ-HWB-9. Therefore, in line with previous research, we used the level sum scores (LSS) of the EQ-HWB-9, ranging from 9 (best health/well-being) to 45 (worst health/well-being) [23]. To minimize respondent burden, the selection of the EQ-HWB-9 was guided by its brevity.

#### Health fluctuation diary

After completing the baseline assessment, caregivers were introduced to the health fluctuation diary and asked to complete it daily for 14 days. A health state fluctuation was defined as any perceived day-to-day change in the PlwD’s overall health compared to the previous day, as judged from the caregiver’s perspective. Caregivers rated the PlwD’s health as (i) much better, (ii) somewhat better, (iii) about the same, (iv) somewhat worse, or (v) much worse (see ​Fig. 1). On day seven, the study nurse reviewed diary entries, provided further assistance to minimize entry errors.

Health fluctuations documented in the daily diary were operationalized in two ways: (i) dichotomously, indicating whether at least one fluctuation was reported during the 14-day period, and (ii) by the total number of fluctuations recorded over 14 days, categorized as no to low (0–5) or moderate to high (> 5) fluctuations.

#### Qualitative semi-structured interview data

After completing the diary and the EQ-HWB-9 on day 14, the study nurse conducted a semi-structured interview with the caregiver, asking about the documented fluctuations and whether these fluctuations were considered in assessing the PlwDs health and well-being using the original “7-DAY” (GROUP 1) or the experimental “TODAY” (GROUP 2) recall period. Caregivers were also asked if they adhered to the respective recall period (today or 7-day) when responding to the EQ-HWB-9.

An interview guide was used, and the study nurses were trained to ensure the replicability of interviews (Supplementary File 2).

### Analyses

Only complete cases were analyzed without imputing missing data. Descriptive statistics were used to describe respondents’ characteristics, health state fluctuations and health changes based on the fluctuation diary and the EQ-HWB-9 Level Sum Scores (LSS). Differences between groups were assessed using ANOVA and Chi^2^ tests. Spearman correlations (r_sp_) were calculated to examine the relationship between self- and proxy-reported LSS at each assessment time point, stratified by recall period.

Based on the LSS, both recall period versions were analyzed descriptively and in terms of variability (variance of the LSS mean change, s2), considering documented health fluctuations from the daily diary entries (no to low fluctuation: 0–5 fluctuations in 14 days, moderate to high fluctuation: more than five fluctuations in 14 days). An absolute mean change ≤ 1 of LSS was considered indicative of low variability, representing a small change relative to the EQ-HWB-9 scale range. Given previous findings demonstrating that greater health fluctuations in PlwD were associated with increased variability in EQ-5D-5 L reports [14], we hypothesized that PlwD with more health fluctuations would show greater EQ-HWB-9 score variability with the today recall than those with low fluctuations using the 7-day recall period. All statistical analyses were carried out using the statistical program STATA 18.

#### Qualitative analysis of recall period adherence

Recall-period adherence was assessed by asking caregivers in the interview whether they had followed the assigned temporal frame (TODAY or 7-DAY) when completing the EQ-HWB-9. This explored whether caregivers understood and applied the intended time period when evaluating the PlwD’s health and wellbeing. This information also provides context for interpreting EQ-HWB-9 results, as deviations from the assigned recall period may affect the health experiences considered when responding to the measure. We dichotomously coded responses (yes/no) and analysed them descriptively by fluctuation frequency. We assessed differences between groups using Fisher’s exact tests and across fluctuation groups using the Chi-squared test. In addition, deviations from the assigned recall period were documented and caregivers were asked what time frame they had considered.

## Results

### Sample characteristics

Seventy-four dyads were included. The sample characteristics are shown in Table 1. PlwD were, on average, 77 years old, primarily male (58%), and moderately cognitively (mean MMST: 18.1) and functionally (mean B-ADL: 6.7) impaired. The two study groups were generally comparable (*p* > 0.05) with regard to demographic (age, gender) and clinical characteristics (MMSE, CCI) at baseline. A statistically significant difference was only observed for B-ADL.

**Table 1:** Patients’ socio-demographic and clinical characteristics and patients’ health-related quality of life at baseline (day 1)

	Overall (*n* = 74)
*Sociodemographics*	
Patient age (mean, SD)	77.3 (8.0)
Caregiver age (mean, SD)	70.6 (10.6)
Female sex patients (n, %)	31 (41.9%)
Female sex caregiver (n, %)	53 (71.6%)
*Relationship*	
Partner/spouse (n,* %)*	58 (78.4%)
Living together (n,* %)*	69 (93.2%)
*Clinical characteristics*	
Deficits in ADL (B-ADL)	6.7 (2.3)
Cognition (MMST)	18.1 (7.9)
Depression (GDS)	6.1 (2.1)
Caregiver burden (Zarit)	8.6 (4.8)
Comorbidity (Charlson)	4.0 (2.1)
*EQ-HWB-9 scores*	
Recall period “7-DAY”	
Patient self-rating LSS, mean (SD, var)	15.1 (4.9, 24.4)
Caregiver proxy-rating LSS, mean (SD, var)	21.5 (5.9, 34.3)
Recall period “TODAY”	
Patient self-rating LSS, mean (SD, var)	14.3 (4.5, 20.1)
Caregiver proxy-rating LSS, mean (SD, var)	19.9 (6.2, 39.3)

*n*= number, SD: Standard Deviation, var: variance, B-ADL: Bayer Activities of Daily Living Scale; MMSE: Mini-Mental State Examination, GDS: Geriatric Depression Scale; Zarit: Caregiver Burden Zarit Interview, Comorbidity: Charlson Comorbidity Index.

Seven dyads had incomplete self- and proxy-reported EQ-HWB-9 data, with no notable demographic differences from complete cases. The mean EQ-HWB-9 self- and the proxy-reported LSS at baseline were 15.1 (SD 4.9, var 24.4) and 21.5 (5.9, 34.3) for the 7-DAY recall period, and 14.3 (4.5, 20.1) and 19.9 (6.2, 39.3) for the TODAY recall period, respectively. Proxy-ratings were higher than self-ratings, and the 7-DAY recall period also yielded higher scores compared to the TODAY recall period, indicating worse health estimates. Correlations between self- and proxy-reported EQ-HWB-9 LSS were weak to moderate across assessment time points. In the TODAY group, correlations ranged from r_sp_=0.36 to 0.45 (each *p* < 0.05), whereas in the 7-DAY group, correlations were weaker at day 1 (r_sp_=0.24), and day 7 (r_sp_=0.14) and moderate at day 14 (r_sp_=0.45, *p* < 0.05).

(Please insert Table 1 here.)

### Health state fluctuations

In total, caregivers (*n* = 74) gave *n* = 908 daily assessments (*n* = 128 missing assessments; 12,4%). Of those, 92% reported at least one daily fluctuation; six caregivers (8%) never reported fluctuations. Caregivers documented health state fluctuations of PlwD in 41.0% of daily assessments (372 of 908), rating PlwDs’ today’s health as better (much: 1.9% or somewhat: 18.2%) or worse (much: 2.2% or somewhat: 18.2%) than the day before. Thirty-six caregivers documented up to five health fluctuations (group: “no to low fluctuation”, while 38 caregivers documented more than five fluctuations (group: moderate to high fluctuation). Health fluctuations are shown in Table 2.

**Table 2:** Frequency of today’s health fluctuation (compared to yesterday) of *n* = 908 daily assessments from 74 caregivers of 74 patients with dementia

Today’s health compared to yesterday	*n* (%)
Much better today	17 (1.87%)
Somewhat better today	170 (18.2%)
About the same today	536 (59.0%)
Somewhat worse today	165 (18.2%)
Much worse today	20 (2.2%)
Σ Fluctuations	372 (41.0%)
Σ No fluctuations	536 (59.0%)

Distribution of the number of health fluctuations	*n* (%)
0	6 (8.11%)
1	4 (5.41%)
2	6 (8.11%)
3	4 (5.41%)
4	10 (13.51%)
5	6 (8.11%)
6	6 (8.11%)
7	12 (16.22%)
8	7 (9.46%)
9	5 (6.76%)
10	3 (4.05%)
11	4 (5.41%)
12	1 (1.35%)

*n *= 128 assessments were missing (1.73 assessments per caregiver)

### Variability of EQ-HWB-9 dimension scores

Across days 1, 7, and 14, EQ-HWB-9 health states changed between assessments in 92.5% of PlwD self-ratings and 100% of proxy-ratings compared with the assessment seven days earlier. Proxy-rated health varied in all cases, irrespective of recall period. Self-ratings varied more frequently with the 7-DAY than with the “TODAY” recall (97.0% vs. 87.9%), however, this difference was not statistically significant (*p* = 0.355).

Across dimensions, exhaustion, sadness/depression, and cognition most frequently changed over time, regardless of perspective. When comparing recall periods at the dimension level, changes were generally more frequent with the 7-DAY recall (Table 3).

**Table 3:** Description of the percentage change of the EQ-HWB-9 dimensions and profile between assessments (day 1 and day 7 or day 7 and day 14)

	Recall period “7-DAYs”	Recall period “TODAY”	*p*-value^1^
*EQ-HWB-9 patient self-rating*	*n* = 33	*n* = 33	
Mobility	33.3%	**51.5%**	***0.010***
Daily activity	45.5%	**51.5%**	0.653
Exhaustion	**75.8%**	69.7%	0.990
Loneliness	39.4%	**45.5%**	0.804
Cognition	**63.6%**	60.6%	0.995
Anxiety	36.4%	30.3%	0.791
Sadness/depression	**75.7%**	69.7%	0.997
Control	42.4%	**51.5%**	0.366
Pain	**45.5%**	30.3%	0.271
Health state	**97.0%**	87.9%	0.355
*EQ-HWB-9 caregiver proxy-rating*	*n* = 34	*n* = 34	
Mobility	**67.7%**	58.8%	0.808
Daily activity	**73.5%**	38.2%	**0.009**
Exhaustion	82.3%	**85.3%**	0.754
Loneliness	**47.1%**	47.0%	0.818
Cognition	73.5%	**79.4%**	0.999
Anxiety	**50.0%**	47.1%	0.999
Sadness/depression	82.4%	**85.3%**	0.999
Control	61.8%	61.8%	0.999
Pain	**50.0%**	44.1%	0.490
Health state	**100.0%**	**100.0%**	0.999
Recall period adherence^**2**^	80%	87%	0.376

Bold indicates a higher percentage change within the respective recall period

^1^Fisher Exact test; ^2^ based on responses of caregivers given during the qualitative, semi-structured interview on day 14 after completing the EQ-5D-5 L., Classification of health fluctuations (frequency): 0–4 low; 5–9 moderate and 9–14 high; percentages and mean change include both self- and proxy responses combined

### EQ-HWB-9 level sum score variation across health fluctuation

Greater diary-documented fluctuation was associated with increased variability in EQ-HWB-9 LSS (Table 4). PlwD with moderate to high fluctuations showed larger absolute changes and greater dispersion than those with no or low fluctuations. Across recall groups, the 7-DAY recall tended to show slightly lower median absolute changes compared with the “TODAY” recall (Table 4, Supplementary 3 Fig. 1). However, distributions overlapped substantially, and isolated extreme values in the 7-DAY group contributed to increased variance.

**Table 4:** Description of patients and caregiver EQ-HWB-9 responses by fluctuation degree (No to low, moderate to high) across recall period groups (“7-DAY” vs. “TODAY”)

EQ-HWB-9 responses	Overall	No to low fluctuation	Moderate to high fluctuation
*“7-DAY” Self-rating*	*n* = 33	*n* = 14	*n* = 19
LSS – day 1, mean (SD)	14.8 (5.6)	12.1 (3.17)	16.9 (6.10)
LSS – day 7, mean (SD)	15.5 (5.7)	12.7 (3.85)	17.6 (6.02)
LSS – day 14, mean (SD)	15.0 (5.2)	11.9 (2.98)	17.4 (5.29)
*Absolute mean (SD) change* ^*1*^	*2.75 (2.86)*	*1.75 (2.16)*	*3.50 (3.14)*
*Median (25%-75% percentiles)*	*2.0 (0.5-4.0)*	*1.0 (0.5-2.0)*	*2.3 (1.5–4.5)*
*Variance (skewness) of abs. change* ^*1*^	*8.22 (1.86)*	*4.68 (1.54)*	*9.86 (1.82)*
*“TODAY” Self-rating*	*n* = 33	*n* = 18	*n* = 15
LSS – day 1	14.6 (4.9)	14.7 (5.47)	14.5 (4.39)
LSS – day 7	13.9 (4.8)	13.1 (3.92)	14.9 (5.87)
LSS – day 14	14.2 (5.4)	13.7 (6.02)	14.9 (4.74)
*Absolute mean (SD) change* ^*1*^	*2.86 (2.04)*	*2.64 (2.06)*	*3.13 (2.06)*
*Median (25%-75% percentiles)*	*3.0 (1.5-4.0)*	*3.0 (0.5–4.5)*	*3.0 (1.5-4.0)*
*Variance (skewness) of change* ^*1*^	*4.19 (0.27)*	*4.26 (0.04)*	*4.27 (0.55)*
*F(df*_*1*_,*df*_*2*_*)*,* p-value*	*1.96*,* 0.048*	*1.10*,* 0.370*	*2.31*,* 0.035*
*“7-Day” Proxy-rating*	*n* = 34	*n* = 15	*n* = 19
LSS – day 1	21.6 (6.2)	19.1 (6.09)	23.6 (5.68)
LSS – day 7	21.9 (6.1)	19.0 (5.80)	24.1 (5.54)
LSS – day 14	21.0 (6.1)	18.3 (6.56)	23.2 (4.79)
*Absolute mean (SD) change* ^*1*^	*2.60 (1.92)*	*2.23 (1.40)*	*2.90 (2.25)*
*Median (25%-75% percentiles)*	*2.5 (1.0-3.5)*	*2.5 (1.0–3.0)*	*2.5 (1.0–4.0)*
*Variance (skewness) of change* ^*1*^	*3.72 (1.68)*	*1.96 (0.71)*	*5.10 (1.57)*
*“TODAY” Proxy-rating*	*n* = 33	*n* = 17	*n* = 17
LSS – day 1	20.7 (6.9)	19.6 (6.11)	21.6 (7.81)
LSS – day 7	20.4 (6.1)	19.8 (6.08)	20.35 (6.72)
LSS – day 14	19.5 (6.4)	18.0 (6.93)	20.29 (6.09)
*Absolute mean (SD) change* ^*1*^	*3.01 (2.08)*	*2.79 (1.57)*	*3.24 (2.52)*
*Median (25%-75% percentiles)*	*2.3 (1.5–4.5)*	*2.0 (2.0-3.5)*	*2.5 (1.5–4.5)*
*Variance (skewness) of change* ^*1*^	*4.32 (1.00)*	*2.47 (0.82)*	*6.34 (0.81)*
*F(df*_*1*_,*df*_*2*_*)*,* p-value*	*1.16*,* 0.290*	*1.26*,* 0.320*	*1.24*,* 0.270*

^1^absolute mean change from day one to day seven and from day seven to day 14; Classification of health fluctuations (frequency): no to low, 0–5 fluctuations within 14 days documented in the diary by a caregiver; moderate to high, more than 5 fluctuations within 14 days; Analysis is based on complete data across all three assessment time points

Furthermore, the proportion of assessments with very low absolute change (≤ 1) was higher in the 7-DAY group (36.4% and 29.4%, respectively) than in the “TODAY” group (24.2% and 20.6%, respectively), particularly among PlwD with no or low health fluctuations (self-assessment: 7-DAY 64.3% vs. 33.5%; proxy-assessment: 7-DAY 33.3% vs. 17.7%) (Supplementary 3 Table 1).

### Adherence to EQ-HWBs’ recall period ​

In total, 73 interviews were conducted; one caregiver was not able to give an interview. The recall period adherence was statistically significant higher in caregivers responding to the recall period of “TODAY” (86%) compared to those responding to the recall period of “7-DAYS” (76%).

For those assigned to the recall period of “7-DAYS”, the adherence was lowest among PlwD without or with low health fluctuations (73.3%), where most caregivers indicated in the interview that the recall period was too long and tended to consider shorter time frames when responding. However, for caregivers of PlwD with moderate to high health fluctuations, recall period adherence increased to 85.0% when using the “7-DAY” recall period. Contrary to this, caregivers responding to a recall period of “TODAY” had the highest recall period adherence in PlwD without or with low health fluctuations (95.0%), but a lower adherence in PlwD with moderate to high fluctuations (76.4%). In these cases, caregivers indicated that the recall period was too short and sometimes considered one or more preceding days, when completing the TODAY assessment. The recall period adherence is demonstrated in Table 5.

**Table 5:** Number and percentage of patients adhering to the original (“7-DAY”) and experimental (“TODAY”) recall period of the EQ-HWB-9, overall and across fluctuation

Caregiver proxy-ratings	Overall	No to low fluctuation^1^	Moderate to high fluctuation^1^
***“7-DAY” recall period***	*n* = 35	*n* = 15	*n* = 20
Number (%) of patients adhering to the recall period	28 (80.0%)	11 (73.3%)	17 (85.0%)
***“TODAY” recall period***	*n* = 37	*n* = 20	*n* = 17
Number (%) of patients adhering to the recall period	32 (86.5%)	19 (95.0%)	13 (76.4%)
***Total sample***	*n* = 72	*n* = 35	*n* = 37
Number (%) of patients adhering to the recall period	60 (83.3%)	30 (85.7%)	30 (81.1%)

^1^absolute mean change from day one to day seven and from day seven to day 14; Classification of health fluctuations (frequency): no to low, 0–5 fluctuations within 14 days documented in the diary by a caregiver; moderate to high, more than five fluctuations within 14 days

## Discussion

### Summary of key findings

With this study, we evaluated the usability of a “7-DAY” vs. “TODAY” recall period in the EQ-HWB-9 for capturing health fluctuations in dementia. It was found that health fluctuations in dementia were common, with health changes reported in nearly half of all daily health diary assessments. The EQ-HWB-9 dimensions most frequently affected by health fluctuations were exhaustion, sadness/depression, and cognition, changing in more than 65% of cases within seven days. The “7-DAY” recall period reflected a broader range of reported problems and tended to capture more variation in these dimensions, but it also showed lower overall variability in absolute score changes, despite occasional outliers contributing to greater score dispersion. Adherence to the assigned recall period was lower when using the “7-DAY” recall period, especially in PlwD without or with only low health fluctuations, with caregivers explaining that the “7-DAY” recall period was too long. However, adherence to the “7-DAY” recall period was high in PlwD with moderate to high fluctuations. In contrast, adherence to the “TODAY” recall period was highest among those with stable health conditions and low among those with frequent health fluctuations. The findings suggest, that the suitability of a recall period may depend on the extent of health fluctuations. This is consistent with the systematic review by Peasgood et al. [8], which found no universally preferred recall period and indicated that preferences for one-day versus seven-day recall may vary depending on symptom variability.

### Health fluctuations in dementia and the use of different administration modes (self vs. proxy)

The high prevalence of health fluctuations observed in our study is consistent with previous research describing dementia as a fluctuation condition characterized by unpredictable day-to-day variations. These are characterized by varying durations of episodes, not only in cognitive dimensions but also in activities of daily living and HRQoL [7, 24–27]. In line with these results, nearly all caregivers in our study reported daily fluctuations in PlwD’s health. The weekly administration of the EQ-HWB-9 confirmed these findings of persistent health changes with changing LSS observed in almost all self- and proxy-ratings, regardless of the recall period used. In a previous study with a similar design [13], day-to-day fluctuations were most commonly associated with changes in mobility, memory, concentration, sleep, and pain/discomfort, as well as usual activities and energy levels. These findings were largely confirmed in the present study.

Beyond the observed health fluctuations, we also identified systematic differences between administration modes (self vs. proxy). Consistent with previous research [11, 28], proxy-ratings were lower than self-ratings, suggesting that caregivers and PlwD may perceive HRQoL differently. This finding further supports the notion that these two raters represent distinct perspectives and should not be considered interchangeable [28]. Taken together, these findings underscore both the high prevalence of health fluctuations in dementia and the challenges in assessing HRQoL in such conditions.

### Effects of recall period

In our analysis, the EQ-HWB-9 scores with a 7-day recall period were lower than those obtained by the EQ-HWB-9 with a recall period of today. Bennett et al. [29] also demonstrated the relevance of recall period, although using a different design: They compared a 7-day retrospective assessment with repeated daily assessments covering the same period and found the 7-day recall to be most concordant with the mean of the daily reports. Our study did not include repeated daily EQ-HWB-9 assessments. Therefore the lower 7-DAY scores observed should not be interpreted in terms of greater or lower accuracy, but rather indicate the temporal frame of assessment may influence the outcome.

Importantly, when examining the observed changes from week to week in EQ-HWB-9 LSS, the mean absolute changes and variance of these changes tended to be lower when the 7-DAY recall period was used, even though this recall period was associated with the occurrence of outliers. The longer recall period, compared with the recall period used today, may capture good or bad days and important health events from the past week, which could result in a health state that differs from the one in the today recall frame. Therefore, the 7-DAY recall period may provide a broader representation of health experiences occurring over the previous week, whereas the “TODAY” recall period may be more closely linked to the individual`s current health state. Given these different temporal perspectives, our findings do not support one recall period as generally preferable but emphasize that its selection and interpretation should consider the characteristics of the condition being assessed. This is consistent with Norquist et al. [30], who emphasized that no recall period is universally optimal and that selection should depend on the concept being measured, disease characteristics, and the intended use of the measure. More recently, Xu et al. [31] compared TODAY (EQ-5D-5L) and LAST WEEK (EQ-HWB-9) recall periods and found small differences in measurement properties. The LAST WEEK version generally captured more reported problems and showed greater informativity, suggesting that recall period choice may influence how health and wellbeing are captured. However, capturing experiences over a longer time frame, may also increase susceptibility to recall bias. This interpretation is consistent with findings from other disease areas. For example Rydén et al. [32], who compared repeated daily symptom reports (RESQ-eD) with a retrospective weekly assessment (RESQ-7) covering the same period in patients with Gastroesophageal Reflux Disease, found that patients slightly overestimated symptom intensity and markedly underestimated symptom frequency on weekly recall. Although their study design differs from ours, these findings illustrate how retrospective integration over a longer recall period may influence reported outcomes.

### Recall period adherence

Regarding adherence to the assigned recall period, most caregivers followed the instructions. However, adherence to the 7-DAY recall period was lower among caregivers of PlwD with no or low health fluctuations. In contrast, adherence to the 7-DAY recall period was higher among caregivers of PlwD experiencing moderate to high levels of health fluctuations. Recent findings by Ng et al. [33] similarly demonstrate that caregivers may not consistently restrict their responses to the specified 7-day recall period and may draw on experiences outside the requested time frame when evaluating the health and wellbeing of PlwD. These suggest that the suitability of a recall period may depend on the fluctuation profile of the individual and support previous research indicating that recall period selection should consider factors such as the purpose of the assessment, its intended use, and the variability of the health condition [34]. Furthermore, future studies may benefit from implementing a clear instructions of the measures purpose to improve participants’ understanding of the assigned recall period [34]. Therefore, recall period selection may involve a trade-off between capturing health experiences over the intended time frame and respondents’ ability to adhere to the assigned recall period, which should be considered when interpreting patient-reported outcomes in clinical and research settings.

### Implications for outcome assessment

Given the challenges associated with selecting an adequate recall period, alternative assessment approaches may warrant consideration. Conducting multiple assessments on consecutive days, using a recall period of today or a weekly basis with a 7-day recall period, so-called momentary data assessments, may be a potential option for more accurately measuring intervention effects when health fluctuates. Blome et al. [35] developed an ambulatory assessment version of the EQ-5D-5L, in which patients with multiple sclerosis rated their health numerous times per day on mobile phones over several days. Patients recommended such daily ambulatory assessments over seven days to assess their own HRQoL most appropriately, which underlines that HRQoL could be measured more accurately in fluctuating health conditions using multiple (ambulatory) assessments. Other studies implemented such strategies in other diseases, such as in rare movement disorders [36]. Future research should examine whether such approaches provide additional information compared with single assessments using longer recall periods, particularly in populations characterized by fluctuating health.

Our findings indicate that recall period selection should be considered in relation to assessment frequency and the expected fluctuation patterns of the target population, as these factors may influence the interpretation of HRQoL outcomes. Although existing recommendations on recall period selection have largely been derived from self-reported outcomes [30, 37], our results suggest these considerations may also apply to proxy-reported HRQoL measures in dementia research. Furthermore, the interpretation of proxy-reported outcomes may also depend on the observability of the assessed health domain. While domains such as mobility and daily activities are more directly observable, subjective experiences such as loneliness, anxiety or sadness may require greater interpretation by caregivers (non-verbal signs) [38, 39]. Building on this, future research should explore hybrid models that combine periodic short-recall assessments with occasional longer-term reflections to balance feasibility and accuracy.

## Limitations

The generalizability of the study findings is limited by several aspects. First, the study design may have influenced the recall behaviour. We did not collect repeated daily EQ-HWB-9 assessments. Therefore, the 7-DAY assessments could not be directly compared with corresponding daily reports covering the same period, limiting conclusions about whether observed differences between recall periods reflect recall effects. We also did not set a recall period as a gold standard to compare it with the other recall period. The missing gold standard can limit conclusions about accuracy, though the study provides evidence on the comparability of recall periods and the implications of recall period selection for outcome assessment. In addition, the intensive daily diary documentation and repeated emphasis on day-to-day health changes could have heightened caregivers’ awareness of fluctuations and encouraged a focus on the current health state. This may have affected adherence to the assigned recall period and potentially led to underestimating non-adherence. Moreover, recall-period adherence was assessed using a study-specific, non-validated question. Although this approach provided insights into how caregivers perceived their application of the assigned recall period, it did not allow us to verify whether they correctly understood or consistently applied the intended time frame. In particular, caregivers may have unintentionally included experiences outside the specified recall period without realizing the deviation. Consequently, the reported adherence rates may overestimate actual adherence and should be interpreted with caution. Furthermore, adherence to the recall periods was assessed only among caregivers, resulting in a one-sided perspective that did not capture views of PlwD. Moreover, repeated questionnaire completion may have introduced a learning effect, potentially influencing how participants interpreted and responded to subsequent assessments. The relatively small sample size (*N* = 74 dyads) limits statistical power. While sufficient to describe health fluctuations, the study was underpowered to detect statistically significant differences between recall groups, particularly regarding score variability. Furthermore, the accuracy of proxy reports may be influenced by the intensity of care provision and the caregiver’s familiarity with the PlwD. However, information on the amount of time spent providing care was not collected in the present study. Our sample included individuals with different dementia subtypes; however, the specific diagnoses were not documented. As patterns of health fluctuations may vary across dementia types and are reported to be more pronounced in Lewy body dementia than in Alzheimer’s disease, we were unable to examine subtype-specific differences. Future studies with larger and diagnostically well-characterized samples are needed to investigate variation in health fluctuations across dementia subtypes. Moreover, an objective clinical assessment of dementia severity was not included in the study. Therefore, the potential influence of disease severity on recall period adherence and HRQoL self and proxy reporting could not be evaluated. Finally, it is not exactly evident to what extent the changes in EQ-HWB-9 scores are caused by random variation instead of manifest health fluctuations. It is essential to ensure that a measure reliably reflects an individual’s health rating, even in the presence of health fluctuations. The EQ-HWB-9 demonstrated satisfactory psychometric performance in previous studies with caregivers [21]. Nevertheless, no additional well-being measure was included in our study, limiting the measures performance in our sample.

## Conclusion

This study provides novel insights into the suitability of two different recall periods (Today vs. 7 Day) when assessing HRQoL in PlwD experiencing recurrent health fluctuations. The findings suggest that recall period preferences and adherence may differ according to an individual’s fluctuation profile, indicating that a single recall approach may not be optimal for all PlwD. These results highlight the importance of considering symptom variability when selecting HRQoL assessment strategies in dementia research. More flexible and context-sensitive approaches may improve the validity of patient-reported outcomes and the accurate representation of patient experiences. Future research should investigate these findings in larger and more diverse samples and explore alternative assessment approaches capable of capturing dynamic changes in health status over time.

## Supplementary Information

Below is the link to the electronic supplementary material.

Supplementary Material 1.

Supplementary Material 2.

Supplementary Material 3.

## Data Availability

Data is available on request.
