# Supplementary material for: Testing the EQ-HWB-9 with seven-day versus today recall periods in dementia: variability and recall adherence under health fluctuations
Source: Qual Life Res. 2026 Nov 6;35(11):320. doi: 10.1007/s11136-026-04432-4 (PMC13743640; doi:10.1007/s11136-026-04432-4)
Supplement: Supplementary file 1 [file 11136_2026_4432_MOESM1_ESM.docx]

**Testing the EQ-HWB-9 with Seven-day vs Today recall periods in Dementia: Variability and Recall adherence under Health Fluctuations**

**Dimensions of the EQ-HWB-9 (EQ-Health and Wellbeing Questionnaire) and level of responses**

**Mobility**

No difficulty

Slight difficulty

Some difficulty

A lot of difficulty

Unable

**Activities**

No difficulty

Slight difficulty

Some difficulty

A lot of difficulty

Unable

**Exhaustion**

None of the time

Only occasionally

Sometimes

Often

Most or all of the time

**Loneliness**

None of the time

Only occasionally

Sometimes

Often

Most or all of the time

**Cognition**

None of the time

Only occasionally

Sometimes

Often

Most or all of the time

**Anxiety**

None of the time

Only occasionally

Sometimes

Often

Most or all of the time

**Sad/depression**

None of the time

Only occasionally

Sometimes

Often

Most or all of the time

**Control**

None of the time

Only occasionally

Sometimes

Often

Most or all of the time

**Pain**

No physical pain

Mild physical pain

Moderate physical pain

Severe physical pain

Very severe physical pain
