# Supplementary material for: Testing the EQ-HWB-9 with seven-day versus today recall periods in dementia: variability and recall adherence under health fluctuations
Source: Qual Life Res. 2026 Nov 6;35(11):320. doi: 10.1007/s11136-026-04432-4 (PMC13743640; doi:10.1007/s11136-026-04432-4)
Supplement: Supplementary file 2 [file 11136_2026_4432_MOESM2_ESM.docx]

**Testing the EQ-HWB-9 with Seven-day vs Today recall periods in Dementia: Variability and Recall adherence under health fluctuations**

**Maresa Buchholz^1^, Lidia Engel^2^, Feng Xie^3,4^, Bernhard Michalowsky^1, 3^**

^1^Patient-reported Outcomes & Health Economics Research German Center for Neurodegenerative Diseases (DZNE), site Rostock/ Greifswald, Ellernholzstrasse 1-2, Greifswald D-17487, Germany

maresa.buchholz@dzne.de

Bernhard.michalowsky@dzne.de

^2^Monash University Health Economics Group, School of Public Health and Preventive Medicine, Monash University, Melbourne, 553 St Kilda Rd, Melbourne VIC 3004, Australia

Lidia.engel@monash.edu

^3^ Department of Health Research Methods, Evidence and Impact, McMaster University, Hamilton, Ontario, Canada

^4^ Centre for Health Economics and Policy Analysis, McMaster University, Hamilton, Ontario, Canada

fengxie@mcmaster.ca

Bernhardmichalowsky@mcmaster.ca

**Contact information for corresponding author**

Dr. Maresa Buchholz

Patient-reported Outcomes & Health Economics Research, German Center for Neurodegenerative Diseases (DZNE), Site Rostock/ Greifswald

Ellernholzstraße 1-2, 17489 Greifswald, Germany

maresa.buchholz@dzne.de

Phone: +49 (0)3834 86 85 32

ORCID: 0000-0002-4486-8632

Word count: 3676

**Funding Statement:**

The EuroQol Research Foundation funded the study (Grand No: 1592-RA). The authors views in the publication do not reflect the views of the EuroQol Foundation.

**Date:**

| **Topic I: Introduction** | |
| --- | --- |
| Dear Mr./Ms. …  I’m glad to be here with you today to talk about the health condition of your relative.  Before we begin, I’d like to briefly explain how the conversation will proceed.  To help me focus better on our discussion, I would like to record the interview. Would that be okay with you?  [If the participant agrees, switch on the recorder now – if not, take notes instead]  Alright, the recorder is now running.  Your participation in this research project is, of course, voluntary. You may stop or pause the interview at any time.  Otherwise, feel free to share as much detail as you feel is appropriate – we have enough time.  Do you have any questions about how the interview will proceed? | - Introduction - Aim of interview - **Recorder** - Voluntariness - Interruption possible - Encourage to talk in detail - **Nachfragen?** |

| **Topic II: Changes in Health** | |
| --- | --- |
| Question 1:  Have there been any changes in your relative’s health over the past two weeks?  If yes, what kind of changes occurred?  [Wait for response]  Did they feel different in any way?  ________________________________________  Question 2:  Were the past two weeks typical of your relative’s usual health status?  [Wait for response]  How was their condition before the study began?  ________________________________________  Question 3:  Were there any particular events that affected your relative’s health during this time?  [Wait for response]  Optional follow-up:  What made this event so significant? | - Diary - Changes in Health - Typical health state? - Special events? |

| **Topic III: The Health Questionnaire EQ-HWB-9** | |
| --- | --- |
| At the beginning and during the study, you completed a questionnaire about your relative’s health. I’ve brought a copy of it with me again today to help you remember it. [Show the participant the questionnaire]  Question 4:  In your opinion, how well does the questionnaire reflect or assess your relative’s health condition?  Is the questionnaire sufficient to capture their health status?  Question 5:  How easy or difficult was it for you to answer the questionnaire? [Wait for response]  Could you tell me a bit more about that? | - **Show EQ-HWB-9** - Difficulties in answering the questions |
| Question 6:  Are some questions more difficult to answer than others? If so, why? / What did you find challenging?  [Wait for response]  Question 7a:  I’ve brought a sheet with me that lists the areas covered by the questionnaire, for example, pain. [Show the sheet and point to the relevant item in the EQ-HWB-S.]  Which of these areas do you think are suitable for assessing your relative’s health condition? You can also point to them on the sheet.  [Wait for response]  Question 7b:  Which areas do you think are missing from the questionnaire to fully capture your relative’s health condition?  [Wait for response]  Do you feel like anything important is missing? | - **Material: Dimensions of the EQ-HWB-9** |

| **Topic IV: Recall-Period** | |
| --- | --- |
| Question 8:  In the questionnaire [point to the questionnaire], you were asked to describe your relative’s health over the past 7 days.  Did you adhere to the "7 days" timeframe when answering all the questions?  [Wait for response]  Why or why not? Or did you consider a longer period when answering the questions?  [Wait for response]  ________________________________________  Question 9:  Now I’d like you to think about your relative’s last 14 days / two weeks.  Do you think that their health over the past 7 days is a good reflection of their overall health during those two weeks?  [Wait for response]  Do the last 7 days represent their condition over the past 2 weeks?  ________________________________________  Question 10:  When you assessed your relative’s health, what timeframe did you actually think about?  In your opinion, what would be an appropriate recall period to best capture their health status? // When you think about your relative’s health, which period do you usually have in mind?  ________________________________________  Question 11:  Health questionnaires are often administered multiple times to get a more accurate picture.  I’d like to know your opinion: how often should this questionnaire be repeated in order to get a reliable understanding of your relative’s health?  [Wait for response] | - Health „7-days“ vs. Longer recall period |
