# Supplementary material for: Testing the EQ-HWB-9 with seven-day versus today recall periods in dementia: variability and recall adherence under health fluctuations
Source: Qual Life Res. 2026 Nov 6;35(11):320. doi: 10.1007/s11136-026-04432-4 (PMC13743640; doi:10.1007/s11136-026-04432-4)
Supplement: Supplementary file 3 [file 11136_2026_4432_MOESM3_ESM.docx]

**Supplementary Figure 1:** Box plots of EQ-HWB-9 absolute changes between days 1 and 7 as well as days 7 and 14

**Supplementary Table 1:** Description of patients and caregiver EQ-HWB-9 responses with an absolute mean change from day 1 to 7 and day 7 to fourteen of less than one by fluctuation degree (No to low, moderate to high) across recall period groups (“7-DAY” vs “TODAY”)

| ***EQ-HWB-9 responses*** | **Overall** | **No to low**  **fluctuation** | **Moderate to high fluctuation** |
| --- | --- | --- | --- |
| ***“7-DAY” Self-rating*** | n=34 | n=15 | n=19 |
| Number (%) of patients with an absolute mean change ≤ 1 | 12 (36.4%) | 9 (64.3%) | 3 (15.8%) |
|  |  |  |  |
| ***“TODAY” Self-rating*** | n=33 | n=18 | n=15 |
| Number (%) of patients with an absolute mean change ≤ 1 | 8 (24.2%) | 6 (33.5%) | 2 (13.3%) |
|  | *p=0.425* | *p=0.153* | *p=1.000* |
|  |  |  |  |
| ***“7-Day” Proxy-rating*** | n=34 | n=15 | n=19 |
| Number (%) of patients with an absolute mean change ≤ 1 | 10 (29.4%) | 5 (33.3%) | 5 (26.3%) |
|  |  |  |  |
| ***“TODAY” Proxy-rating*** | n=34 | n=17 | n=17 |
| Number (%) of patients with an absolute mean change ≤ 1 | 7 (20.6%) | 3 (17.7%) | 4 (23.5%) |
|  | *p=0.413* | *p=0.424* | *p=1.000* |

^1^absolute mean change from day one to day seven and from day seven to day 14; Classification of health fluctuations (frequency): no to low, 0-5 fluctuations within 14 days documented in the diary by a caregiver; moderate to high, more than 5 fluctuations within 14 days
